# Diagnostic Techniques and Epidemiological Methods for Parasites in Beekeeping: Considerations and Perspectives

**DOI:** 10.3390/pathogens15010084

**Published:** 2026-01-12

**Authors:** Roberto Bava, Fabio Castagna, Stefano Ruga, Rosa Maria Bulotta, Giovanna Liguori, Domenico Britti, Ernesto Palma, Vincenzo Musella

**Affiliations:** 1 Department of Health Sciences, University of Catanzaro Magna Græcia, 88054 Catanzaro, Italy; 2 Local Health Authority (ASL) Foggia, 71121 Foggia, Italy

**Keywords:** *Apis mellifera*, *Varroa destructor*, *Vairimorpha* spp., *Acarapis woodi*, *Tropilaelaps* spp., *Lotmaria passim*, *Crithidia mellificae*, parasitological diagnosis

## Abstract

Pests contribute significantly to the loss of *Apis mellifera* colonies in a multifactorial context that includes viruses, pesticides, nutritional deficiencies, and climate change. This review critically summarises diagnostic techniques (morphological, molecular, automated) and epidemiological methods for the main parasites (*Varroa destructor*, *Vairimorpha* spp., *Acarapis woodi*, *Tropilaelaps* spp., *Aethina tumida*, *Lotmaria passim*, *Crithidia mellificae*), evaluating trade-offs between sensitivity, specificity, cost, and practicality. There is no universal gold standard; the methodological choice must be contextualised. A decision-making framework structured on four pillars (Primary objective, Resource constraints, Epidemiological context, Ethics/Regulatory) is proposed to guide optimal selections, with application examples and testable hypotheses for future validation. Limitations of emerging technologies (reduced accuracy in the field for AI and LAMP), gaps in multi-pathogen synergies (including viruses and bacteria), interactions with pesticides, and climate impacts with explicit uncertainties are discussed. A global perspective and a One Health approach are adopted, identifying research priorities for integrated diagnostic tools, validated predictive models, and sustainable strategies.

## 1. Introduction

Global beekeeping faces complex and multifactorial health challenges, with colony losses in some regions consistently exceeding 30–40% annually and even higher peaks during critical periods.

These data, initially documented by van Engelsdorp et al. (2008) [1] and Neumann and Carreck (2010) [2], have been confirmed and updated by recent surveys reporting that losses reached 55–62% of managed colonies in the United States between April 2024 and April 2025 [3], with values exceeding 60% among commercial operators [4,5].

The associated economic impacts are substantial, estimated at approximately USD 600 million for the US alone, with knock-on effects on agricultural pollination and food security [6].

Pests constitute one of the most important contributing factors within this scenario, acting in concert with viruses, pesticide exposure, nutritional deficiencies, and environmental changes [7].

In particular, *Varroa destructor* is recognised as a key driver of colony losses globally, mainly due to its role as a vector of pathogenic viruses (such as deformed wing virus, DWV, one of the most clinically significant and widely studied) and for amplifying the effects of other stresses [8].

Consequently, knowledge of diagnostic techniques and a correct epidemiological classification of parasites are fundamental to understanding and mitigating these losses [9]. However, applying these concepts in beekeeping encounters unique obstacles due to the sector’s specific parameters and the complex social organization of honey bees.

Epidemiology, traditionally defined as the study of disease distribution and determinants in populations, has required significant adaptation to address the peculiarities of the honey bee colony, a “super-organism” where the epidemiological “individual” can vary from a single insect to the entire colonial unit [10,11].

This conceptual complexity, explored in detail by van Engelsdorp and colleagues (2013) [11], requires a rethinking of classical epidemiological categories and the adaptation of diagnostic methodologies capable of operating effectively at multiple organisational levels simultaneously.

While standardized methodologies for the diagnosis and epidemiological study of key parasites (*Varroa destructor*, *Vairimorpha* spp. (*Vairimorpha* spp.), *Acarapis woodi*, and emerging parasites such as *Tropilaelaps* spp., *Aethina tumida*, *Lotmaria passim*, and *Crithidia mellificae*) exist, a critical gap remains between methodological availability and effective, context-specific implementation.

The unresolved problem lies in the absence of a pragmatic framework to guide the optimal selection of diagnostic and epidemiological approaches, which must navigate trade-offs in sensitivity, specificity, cost, and practicality without a universal gold standard.

To address this gap, this review has three defined objectives.

The first is to provide a critical and up-to-date overview of standardized methodologies for diagnosing and studying the main bee parasites, systematically analyzing the advantages, limitations, and trade-offs of morphological, molecular, and automated techniques.

The second is to propose a novel decision-making framework, structured on four interdependent pillars (Primary Objective, Resource Constraints, Epidemiological Context, and Ethics and Regulatory Aspects) designed to guide optimal methodological selection tailored to specific purposes and operational realities.

The third is to highlight principal knowledge gaps, including the quantitative understanding of multi-pathogen synergies, interactions with pesticides, and climate change impacts, which constitute key research priorities.

All of this is intended to lead to a better standardization of diagnostic techniques. Methodological standardisation is essential for enabling reliable comparisons across studies conducted in different locations, management conditions, and geographical contexts, thereby supporting evidence-based health management policies [12].

This review aims to contribute a practical, decision-oriented synthesis that moves beyond generic recommendations. Its primary novelty is the proposed framework, derived from veterinary diagnostic principles but adapted for beekeeping, which favors transparent and replicable choices that can be empirically validated in future trials.

## 2. Literature Search and Review Methodology

This manuscript constitutes a comprehensive narrative review. Its methodology was designed to systematically identify and critically evaluate the existing scientific literature concerning diagnostic and epidemiological methods for key honey bee parasites.

A structured literature search was conducted to map the available evidence, querying the electronic databases PubMed, Scopus, and Web of Science (2000–2024). The core search strategy employed the Boolean string: (*Apis mellifera* or honey bee) and (diagnosis or detect or monitor) and (*Varroa destructor* or *Vairimorpha* or *Acarapis woodi* or *Tropilaelaps* or *Aethina tumida* or *Lotmaria passim*).

The study selection was guided by specific criteria. Studies included were primary research articles or reviews in the English language focusing on diagnostic techniques and/or epidemiological study designs for the specified parasites in *Apis mellifera*. Conversely, studies were excluded if they focused solely on treatment efficacy without diagnostic components, pertained to other bee species without direct relevance, or were non-peer-reviewed literature.

The screening process involved two primary stages. Initially, the titles and abstracts of all retrieved records were assessed for relevance by one author. To ensure consistency, a subset representing approximately 20% of these records was independently screened by a second author, with any discrepancies resolved through discussion. Following this, the full texts of all potentially relevant studies were obtained and evaluated against the inclusion criteria.

The final corpus of literature was determined by its relevance to the review’s objectives and the availability of evidence. Furthermore, key authoritative reports from major apicultural networks and agencies, including COLOSS, EURL, and USDA-ARS, were manually incorporated to ensure comprehensive coverage of critical technical guidelines and surveillance data.

The subsequent analysis was qualitative and comparative in nature. The selected body of literature was critically examined to synthesize the underlying principles, advantages, limitations, and practical trade-offs associated with the different diagnostic and epidemiological approaches discussed.

## 3. The Hive’s Parasite

### 3.1. Varroa destructor

The impact of *Varroa destructor* on *Apis mellifera* colonies is among the most significant factors in global colony losses, mainly due to its role as a parasitic mite and vector of pathogenic viruses, with deformed wing virus (DWV) being the most clinically significant and widely studied) [13].

This mite acts synergistically with other stresses (viruses, pesticides, poor nutrition), amplifying the overall effects on colony health [14]. Regular monitoring is an essential practice in modern beekeeping, adapted to different regional and management contexts, including those with tolerant populations (e.g., Africanized bees in the Americas) [15].

The biology of the mite presents a dichotomy between the phoretic phase (on adult bees, feeding on fat body and haemolymph) and the reproductive phase (exclusively in capped brood cells, with a preference for drone brood) [16].

This characteristic implies that the diagnostic method chosen determines the type of information obtained: the immediate parasitic pressure on the adult population or the potential for future growth of the infestation.

Effective diagnosis, therefore, requires a clear definition of the objective, integrating complementary methods for a complete picture.

The main methods used to quantify mites in the phoretic phase are washing with ethanol (or soapy water) and shaking with icing sugar [17]. Washing is considered the gold standard method, with sensitivity close to 98–100%, under controlled conditions, though field variability can reduce it slightly.

The standard protocol involves sampling approximately 300 worker bees (preferably from brood combs), immersing them in cleaning fluid, shaking vigorously, and filtering for accurate counting. The advantages include high accuracy and the possibility of storing samples for further analysis, but the main disadvantage is the lethality for the sampled bees and the need for reagents, limiting its use in extensive or repeated monitoring [17,18].

The powdered sugar method is a non-lethal and practical alternative: a similar sample of bees is shaken into a jar of sugar, which dislodges the mites, causing them to fall through a mesh. The bees can be returned to the hive, reducing the impact on the colony. Sensitivity is variable (75–90%), influenced by operational factors (humidity, temperature, operator experience) [17,18].

Recent developments in automation, such as image recognition-based smartphone applications, achieve >90% accuracy under controlled conditions, but drop to ~80–85% in the field due to environmental variables (e.g., wet bees, debris) [19,20,21]. These tools are promising for rapid screening but require confirmation with traditional methods for critical decisions (Table 1).

An essential complementary approach is direct examination of capped brood, which provides information on mite reproduction. By opening 200–300 cells (preferably drone cells, which are more attractive), the intra-colony prevalence (i.e., percentage of infested cells) and intensity (average mites per infested cell) can be determined [18].

This method is more invasive and laborious but provides robust estimates of total population and reproductive effectiveness, which are crucial for predictive models of infestation growth [18].

For continuous, non-invasive monitoring, natural falls on mesh flooring remain useful: by counting mites that have fallen daily/weekly, temporal trends can be obtained. Interpretation requires empirical correction factors, which vary depending on the presence of brood (from 20–40 without brood to 250–500 with active brood), introducing significant uncertainty [18].

In addition to counting, modern diagnostics include assessment of resistance to acaricides, which is no longer optional given its global spread. A distinction is made between field efficacy tests (post-treatment population reduction) and laboratory tests (e.g., vial tests for sensitivity). Molecular analyses (search for mutations in target genes, e.g., sodium channel) allow early identification and tracking of resistance [15].

Data integration is achieved through dynamic intervention thresholds: the classic 3% phoretic threshold (3 mites/100 bees) in late summer is a useful guide in temperate climates, but must be adapted to brood presence, host genetics, viral load (DWV as a driver of collapse), and regional context [22].

From a global perspective, tolerant populations (e.g., Africanized) show higher thresholds without collapse. The use of Geographic Information Systems (GIS) to map infestations and resistance allows for the identification of risk clusters and coordinated territorial strategies [23].

### 3.2. Vairimorpha spp.

Following recent phylogenetic reclassification, *Nosema* spp. infecting honey bees are now placed in the genus *Vairimorpha* [24]. The diagnosis of *Vairimorpha* spp. infections is a significant example of how advances in knowledge about pathogenicity and species diversity have driven progress in diagnostic practices [25].

Historically, nosemosis was mainly attributed to *Nosema apis*, with monitoring based on spore quantification. The discovery and global spread of *Nosema ceranae* (morphologically similar but with potentially relevant differences in terms of seasonality, pathogenicity, and response to treatment) has necessitated methodologies capable of distinguishing between the two species [26,27]. Today, diagnosis is not limited to quantifying the parasite load (“how many spores”) but must prioritise identifying the dominant species (“which spores”), especially in contexts of frequent co-infections and synergies with other intestinal pathogens (e.g., *Lotmaria passim*) [28].

The traditional and still widely used method for initial quantitative assessment is spore counting in a Neubauer chamber. The standard protocol involves homogenising a sample of 30–60 bees (preferably foragers, with higher loads) in distilled water, followed by transferring a drop of the suspension to a haemocytometer. Observation at 400× magnification using a phase contrast optical microscope allows the refractive spores to be counted and the average number per bee to be calculated [29]. This approach is accessible (requiring basic equipment and minimal reagents), rapid, and provides a useful semi-quantitative estimate for routine monitoring. However, it does not allow a reliable distinction between *Vairimorpha apis* (slightly larger spores, ~5–6 × 3 µm, uniform oval shape) and *Vairimorpha ceranae* (spores ~4–5 × 2–3 µm, often more curved; range in literature Fries, 1993) [29]. Furthermore, it does not discriminate between viable and non-viable spores, potentially overestimating the infectious risk [29].

To overcome these limitations, molecular diagnostics have become essential. Conventional PCR amplifies specific regions of ribosomal DNA (e.g., 16S rRNA), producing fragments of different lengths for the two species, which can be visualised on agarose gels. This method offers reliable qualitative identification of the presence of *Vairimorpha apis*, *Vairimorpha ceranae*, or co-infections. A key advancement is real-time qPCR, which quantifies the genomic load with high sensitivity (~10^1^–10^2^ spore equivalents/bee) and allows precise correlations with physiological parameters or treatment efficacy [29]. However, the conversion from genomic copies to viable spores is not linear, and there is a risk of false positives from residual DNA from dead spores.

Access remains limited by cost and the need for specialised laboratories. A promising compromise is represented by isothermal amplification techniques, such as LAMP (Loop-Mediated Isothermal Amplification). Commercial kits operate at a constant temperature (~63 °C) in a simple thermostatic bath, producing visible results (cloudiness) in 40–60 min, with low costs and high specificity. These tools are facilitating species-specific diagnosis in non-laboratory settings (veterinarians, beekeepers, associations), but they are sensitive to inhibitors in complex samples and limited in advanced multiplexing [30,31].

Transmission electron microscopy remains the morphological gold standard for ultrastructural details (e.g., polar filament coils: >30 in *Vairimorpha apis*, 20–23 in *Vairimorpha ceranae*) but is reserved for taxonomic research or controversial cases due to its complexity and cost [29].

The choice of method should be guided by the specific objective. For regional surveillance or large-scale seasonal monitoring, microscopic counting on pooled samples remains efficient. For investigations of atypical depopulations, synergistic interactions, or therapeutic trials, qPCR or multiplex panels (including *Lotmaria passim*) are indispensable for a complete aetiological picture (Table 2). There is also a need to consider false positives in qPCR and pathogenicity variability: *Vairimorpha ceranae* shows more variable effects than initially assumed, with subtle impacts on physiology and immunity rather than direct mass mortality [32]. The interpretation of results requires integration with epidemiological patterns: *Vairimorpha apis* shows spring peaks linked to winter confinement, while *Vairimorpha ceranae*, which is more thermophilic, may show more uniform distributions or summer peaks, with geographical variations (e.g., higher prevalence in Mediterranean climates) [33,34]. A high burden of *Vairimorpha ceranae* in summer has different prognostic implications than in spring. In global contexts, regional differences (e.g., lower impact in some adapted populations) underscore the importance of local data for management decisions.

### 3.3. Acarapis woodi

*Acarapis woodi*, the bee tracheal mite, occupies a special place in modern apiculture pathology: historically considered a significant threat, its impact has diminished in many regions thanks to the widespread use of synthetic acaricides to control *Varroa destructor*, many of which have a suppressive side effect on this parasite [35]. However, with the increasing adoption of integrated management practices and the reduction in the use of certain active ingredients (to counter resistance or for biological preferences), a resurgence could emerge in specific contexts, especially in climates with prolonged winters. This mite completes its entire life cycle in the thoracic tracheal system of adult bees, feeding on haemolymph and causing mechanical damage to the respiratory structures [36]. Clinical symptoms are often non-specific (general weakness, reduced flight capacity, high winter mortality) and may include wing misalignment (K-wing), more commonly associated with interactions with viruses or other stresses than with the mite alone [36,37].

The diagnosis of *Acarapis woodi* is closely linked to its biology, which imposes specific constraints on sampling. The mite has a marked specificity for the age of the host: fertilised females mainly transfer to very young bees (<4–9 days old), when the cuticle is still permeable [36]. After this window, sclerotisation makes the bee resistant to invasion. Drones can host more intense infestations due to their larger tracheal diameter, but workers are the main reservoir [36]. Consequently, random sampling of foragers (older bees) is unlikely to detect infestations; sampling should target young bees from the central combs of the nest, ideally in late autumn or early spring, when prevalence reaches seasonal peaks.

The diagnostic method of choice remains direct morphological examination through dissection. The classic protocol (Milne, modified [38]) involves fixing the bee under a stereomicroscope, removing the head and first pair of legs, extracting the scleritic “neck”, and dissecting the main thoracic tracheae for observation at high magnification [39]. This approach is sensitive but laborious (15–30 min per bee) and risks losses during handling. Improved variants include the thoracic flap method [40], which allows in situ observation without complete extraction, and the thoracic disc method (TDM), with clarification in hot KOH to make tissues transparent and visualise mites inside the tracheae without invasive dissection.

Given the laborious nature of morphological methods, molecular approaches have been developed for more scalable screening. Conventional and nested PCR protocols amplify specific sequences (e.g., mitochondrial cox1 gene), detecting mite DNA from thoracic homogenates without dissection [39]. qPCR with TaqMan probes achieves high sensitivity (equivalent to 1–2 mites or prevalences < 2% in pools), facilitating large-scale surveillance [41].

These methods have not yet completely replaced microscopy in routine practice, but they are valuable for rapid confirmation or in reference laboratories, especially in low-prevalence settings. Diagnostic interpretation requires contextualisation: prevalences < 20–30% are often subclinical, especially in mild climates, while >40–50% increase the risk of winter mortality in cold regions [42]. The decline observed in Europe is mainly attributed to anti-*Varroa* treatments, suggesting that apiaries with organic management or low dependence on synthetic acaricides deserve specific monitoring for possible recovery. Globally, *A. woodi* remains relevant in areas with harsh winters (North America, parts of northern Europe), while it is less problematic in tropical or subtropical climates [43].

The diagnosis of tracheal acariosis should be considered in cases of spring weakness not explained by other primary factors, especially in historic apiaries or those transitioning to sustainable practices. Integration with multiplex molecular panels (including respiratory viruses) could improve understanding of interactions and guide evidence-based management decisions.

## 4. Emerging Parasite in Europe and Worldwide

Pests considered emerging or invasive in many regions (*Tropilaelaps* spp., *Aethina tumida*, *Lotmaria passim*, and *Crithidia mellificae*) pose distinct diagnostic challenges compared to established endemic pests such as *Varroa destructor*. The primary objective is not so much the routine quantification of a stable pest load, but rather early detection, reliable identification, and management of the risk of introduction or expansion, often in a context of territorial biosecurity and climate adaptation [44,45]. These pathogens share the need for hierarchical approaches: broad, low-cost initial screening, followed by specialised confirmation and, where required, molecular taxonomic identification. Synergies with endemic parasites or viruses further amplify the relevance of integrated diagnostics.

### 4.1. Tropilaelaps spp.

The genus *Tropilaelaps* is one of the most serious invasive threats to global beekeeping outside its native Asia [46]. The main species (*T. mercedesae*, *T. clareae*, *T. koenigerum*, *T. thaii*) [47] are obligate brood parasites with a very short phoretic phase (<3 days) [48], which makes them potentially more devastating than *Varroa* if they become established. In Asia, frequent co-occurrence with *Varroa* complicates differential diagnosis and implies competitive or synergistic interactions that are not fully understood [49].

Biology imposes stringent diagnostic constraints for surveillance in disease-free areas: methods based on adult bees (ethanol washing, powdered sugar) are severely undersensitive, with a low probability of detection even in heavy infestations. The effective approach is destructive sampling of capped brood: uncapping at least 100–200 cells (preferably drone cells), removing pupae/larvae, and tapping the comb on a white surface to observe mobile mites (light brown, elongated, ~1 mm, rapid movement) [50]. Targeted visual inspections of the combs complete the screening, looking for indirect signs (irregular brood, mutilated larvae).

Once suspected, morphological identification distinguishes *Tropilaelaps* from *Varroa* (more elongated body, light colour, high mobility) [51], but species-specific determination requires advanced microscopic preparation (clarification in Hoyer’s medium, observation of chelicerae or copulatory organs) [52]. Molecular techniques are the standard for confirmation: PCR on mitochondrial markers (cox1) or ITS, followed by sequencing or PCR-RFLP for rapid, distinctive patterns [52]. This identification is not a mere taxonomic exercise: different species show variations in adaptability to *Apis mellifera* and invasive potential (*T. mercedesae* is considered the riskiest).

Recent bioclimatic models indicate a possible expansion of climatic suitability in southern/central Europe of 30–50% by 2050 under SSP2-4.5 scenarios, with uncertainties related to model variability and winter brood interruptions [53]. Surveillance protocols should integrate routine visual inspections (by trained beekeepers/technicians), targeted sampling in risk areas (ports, imports), and molecular confirmation in reference laboratories for the activation of emergency plans.

### 4.2. Aethina tumida

*Aethina tumida*, native to sub-Saharan Africa (where it coexists in balance with local bees), becomes highly destructive in new regions due to the absence of effective host defences. Introduced in the USA (1996), Australia, and Europe (Italy 2014; Portugal), the diagnosis focuses on early detection for containment [54,55].

The cycle (adults in the hive, destructive larvae on brood/honey/pollen, pupation in the soil) guides dual strategies: hive and apiary. Morphological identification is key: adults (5–7 mm, brown-black, distinctive antennae, rapid escape from light); larvae (~10 mm, cream-coloured, three pairs of developed thoracic legs, characteristic dorsal spines, key differentiation from *Galleria mellonella*) [56,57]. Indirect signs are represented by fermented honey (rotten citrus smell), tunnels in honeycombs.

Surveillance includes visual inspection (nest corners, honeycomb covers, side frames) and specific traps (e.g., Beetle Blaster with oil): dual tools for early monitoring and mechanical control [58]. Climate models predict expansion of suitable areas in the Mediterranean with rising temperatures, making sentinel trapping essential in coastal regions.

### 4.3. Lotmaria passim and Crithidia mellificae

Intestinal trypanosomatids have undergone an epidemiological shift: *Lotmaria passim* has become dominant in Europe (>70–90% prevalence in many areas since 2016–2017, particularly in Europe, with lower rates in Asian and African contexts) [59], replacing *Crithidia mellificae*. *L. passim* alters the intestinal microbiome (reduction in *Lactobacillus/Bifidobacterium* > 60%), compromises learning/olfactory memory, and increases susceptibility to *Vairimorpha ceranae* [60,61,62]. Co-infection synergies show variable effects: recent analyses estimate increased winter mortality when *L. passim* is associated with *Vairimorpha ceranae* [61].

Morphological diagnosis (observation of flagellates in intestinal smears) is limited and non-discriminatory; the gold standard is species-specific PCR on 18S rRNA or ITS regions [63]. Multiplex panels (simultaneous detection of *Lotmaria*, *Vairimorpha apis/ceranae*) optimise costs and provide a complete aetiological picture for intestinal disorders.

In the sampling phase, it is recommended to use foraging bees or, alternatively, to dissect the intestine. This reduces the amount of contaminants and inhibitors (such as pigments or enzymes) that could compromise the reliability of the subsequent molecular test (PCR).

The absence of authorised treatments in Europe shifts management to resilience support (optimal nutrition, stress reduction). *C. mellificae* remains secondary, relevant mainly in competition studies.

## 5. Interactions Between Emerging Parasites

The health of the hive is compromised not only by individual pathogens, but increasingly by complex multi-parasite interactions that amplify damage synergistically. Co-infections are the norm rather than the exception, and their integrated diagnosis is crucial for a realistic understanding of risk. The most studied interaction is that between *Lotmaria passim* and *Vairimorpha ceranae*, where the flagellate, by altering the intestinal microbiome and immune barrier, significantly increases the pathogenicity of microsporidia [61]. Recent studies quantify this impact, showing how co-infection alters the foraging behaviour and physiology of bees, reducing return rates to the hive and accelerating population turnover [61]. Similarly, *Varroa destructor* acts as a vector and activator of viruses, particularly Deformed Wing Virus (DWV) [64]. Mite infestation, especially under conditions of heat stress induced by climate change, increases viral replication and leads to lethal viral loads, driving colony collapses [65]. These synergies require a diagnostic paradigm shift: from single-target panels to multiplex molecular panels capable of simultaneously detecting key parasites and viruses. Only a complete aetiological profile allows the total pathogen load of a colony to be assessed and management strategies to be directed towards strengthening overall resilience rather than controlling a single agent. It is important to note that while these synergistic relationships are strongly supported by correlative and experimental data, the direction of causality and the influence of confounding environmental factors (e.g., concurrent pesticide exposure, overall colony nutrition) require further elucidation in field settings.

## 6. Integrated Epidemiological Approach

Bee epidemiology deals with a multifactorial reality in which diseases do not derive from a single causative agent but from the dynamic interaction between parasites, viral pathogens, environmental factors, management practices, and nutritional stress [66]. This requires an integrated approach that combines diverse study designs, advanced statistical analyses, and spatial tools to generate robust evidence that can be translated into operational practices. The aim is not only to describe the distribution of diseases, but also to identify causal relationships, quantify risks, and evaluate interventions, taking into account the hierarchical structure of beekeeping data (bees within colonies, colonies within apiaries, apiaries within regions).

### 6.1. Types of Epidemiological Studies

The choice of study design must balance the strength of evidence, available resources, and ethical issues. Cross-sectional studies provide an efficient snapshot of prevalence and associations, ideal for large-scale surveys such as COLOSS, but do not establish temporal causality [67]. Prospective cohort studies, following colonies over time, provide robust relative risk measures, but are costly and subject to follow-up losses. Retrospective case–control studies are efficient for rare factors (e.g., acaricide resistance), but sensitive to recall bias, i.e., bias due to differences in memory ability or accuracy between the groups being compared [11]. Finally, randomised controlled trials (RCTs) represent the gold standard for testing interventions (e.g., new anti-*Varroa* treatments or nutritional supplements), with randomisation minimising confounding factors, although they are challenging in terms of blinding and costs in beekeeping [11]. The main characteristics of different epidemiological study designs are summarized in Table 3.

### 6.2. Sample Size and Statistical Power

Power calculations are essential to avoid false negatives or waste of resources. To give an idea, even under ideal conditions estimating a prevalence of 5% with an accuracy of ±2% (95% CI) would require a sample of about 456 colonies, assuming simple random sampling.

However, in beekeeping reality the cluster structure (related bees within the colony, related colonies in the apiary) greatly increases this number: Typical intraclass correlation coefficients (ICCs) (0.1–0.3) dictate that the sample size should be multiplied by 20–50% or more, depending on the number of colonies per apiary.

Tools such as cluster sampling formulas or software (e.g., R package “survey”) facilitate these calculations, also incorporating design effects for nested apiaries [11].

### 6.3. Hill’s Criteria for Causal Inference

Observed association (e.g., pesticides and colony losses) does not imply causality. For this reason, in beekeeping epidemiology, the Hill criteria [68] are used as a compass. It is evaluated, for example, if the association is strong and constant in different studies (meta-analyses), if the exposure is clearly prior to the damage, if the outcome worsens as the ‘dose’ increases (such as the varroa load), if the mechanism is biologically plausible (e.g., transmission of viruses) and if there are confirmations from controlled experiments.

Critically applied, they highlight that *Varroa* is a primary causal factor, while pesticides/nutrition act as risk modifiers in synergistic contexts.

### 6.4. Multivariate Analysis and Complex Models

Multivariate models address collinearity among factors. Binomial logistic regression (outcome: colony survival) quantifies adjusted odds ratios. Hierarchical mixed models incorporate random effects for apiary/colony, reducing bias from clustering.

Techniques such as random forests or CART capture non-linear interactions (e.g., *Varroa* × nutrition × climate) [69].

## 7. A Decision Framework for Diagnostic and Epidemiological Method Selection

The proliferation of diagnostic techniques and epidemiological approaches for apistic parasites, while testifying to the vigorous scientific progress of the sector, risks generating a paradox: an excess of choices that can paralyze the researcher, technician, or beekeeper.

The question is no longer simply “with which method can I detect parasite X?” but “which, among all available methods, is the most appropriate for my specific purpose, in my specific context, with my specific resources?”

To answer this complex question, it is necessary to abandon generic recommendations and adopt an explicit and systematic decision framework, based on transparent and replicable criteria.

**Figure 1:**
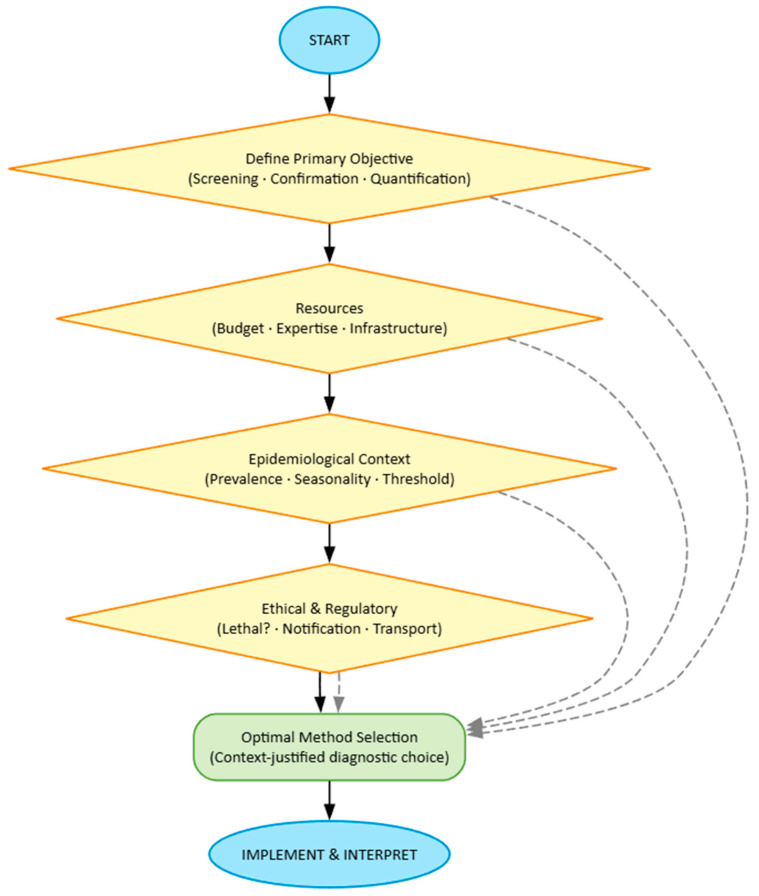
Flowchart illustrating the four interdependent pillars that guide the optimal selection of diagnostic methods for honey bee parasites. The process involves sequential consideration of each pillar’s criteria to converge on a contextualized and justified choice.

We propose here such an algorithm, structured on four interdependent pillars. This framework, structured on four interdependent pillars, is visualized in Figure 1. The following sections detail each component.

The first pillar concerns the clear definition of the Primary Objective. This is the compass of the entire decision-making process. Methodological choice diverges radically depending on whether one aims for rapid and extensive screening (where cost per sample and speed are paramount), diagnostic confirmation in a suspected case (where specificity and positive predictive value are crucial), or precise quantification for dose–response or therapeutic efficacy studies (where accuracy and repeatability are essential). Similarly, it is fundamental to distinguish whether the unit of interest is the individual (e.g., pathogenesis studies) or the population (colony, apiary, region). A perfect method for estimating individual *Vairimorpha* load (such as qPCR on a single bee) would be prohibitive and inefficient for estimating prevalence in a region, where pooled sampling and optical microscopy might offer the best information-to-cost compromise.

The second pillar imposes a realistic examination of Resource Constraints. Ideal science exists in a vacuum of infinite budget and cutting-edge equipment; applied science does not. Cost per sample must include not only reagents but also personnel time, shipping expenses, and equipment amortization. Available technical expertise is an often underestimated filter: a qPCR protocol requires competencies and laboratory quality control absent in field contexts. The infrastructure itself (availability of phase-contrast microscope, thermocycler, −80 °C freezers) defines the perimeter of the possible. Ignoring these constraints leads to unsustainable protocols that fail in their practical implementation.

The third pillar requires contextualizing the choice within the specific Epidemiological framework. A method’s effectiveness depends intrinsically on the field situation. Expected disease prevalence conditions the necessary sample size and test predictive value: in a high-prevalence population, even a test with moderate specificity can be useful, while in a low-prevalence population, only tests with very high specificity avoid an excessive proportion of false positives. The expected infestation level relative to the clinical or intervention threshold is critical: methods with low sensitivity (such as visual monitoring for *Varroa*) are entirely inadequate for detecting low but indicative infestations of future growth, while they may be sufficient when infestation is already clinically manifest. Parasite seasonality influences sampling plans and matrix choice (adult bees vs. brood).

The fourth pillar integrates Ethical and Regulatory dimensions. Apistic research cannot ignore these considerations. Acceptability of lethal methods is both an ethical and practical question: destructive sampling is justifiable for a definitive trial on drug efficacy, but not for routine monitoring in a conservation apiary. Mandatory notification requirements for certain parasites (e.g., *Tropilaelaps*) may influence confirmation method choice, pushing toward protocols that produce incontrovertible evidence accepted by authorities. Restrictions on biological sample movement, finally, may determine whether analysis must be conducted in the field (with point-of-care kits) or in a centralized laboratory.

### Practical Application: The Case of Varroa destructor

The utility of this integrated framework is exemplified by application to the most studied parasite:

1. Scenario: National Monitoring (Objective: Spatial Trends; Constraints: Limited Budget, Non-Specialized Personnel). The objective is to collect comparable data on a large scale to map risk. Absolute precision on a single colony is sacrificeable in favor of geographic coverage. The optimal choice falls on the powdered sugar method (low cost, non-lethal, executable by technicians after brief training), whose data, despite their uncertainty, integrated into a Geographic Information System (GIS), allow identification of spatial clusters and reliable regional trends.
2. Scenario: Evaluation of a New Acaricide in Controlled Trial (Objective: Absolute Accuracy and Quantification; Constraints: High Budget, High Expertise, Equipped Laboratory). Here, the objective is to measure often subtle differences in efficacy. The gold standard is required: ethanol wash offers maximum accuracy in phoretic mite counting. To investigate mechanisms of action (e.g., effects on reproduction), this can be associated with destructive brood sampling and qPCR techniques to evaluate gene expression in surviving mites. The cost and ethics of the method are justified by the strength of evidence required.
3. Scenario: Professional Beekeeper (Objective: Timely Treatment Decision; Constraints: Limited Time, Low Cost, Practicality). The objective is to apply an intervention threshold. A continuous non-invasive monitoring system (screened bottom board with automatic app counting) provides a real-time trend with minimum disturbance, alerting when the threshold is exceeded. It is the quintessence of a “fit-for-purpose” method: it does not provide the most precise absolute count but the operational information necessary for a correct and timely management decision.

This rational approach, which abandons the single recipe to embrace contextual choice, finds its most advanced expression in integrated and hierarchical diagnostic strategies. The future does not lie in the uncritical adoption of the latest technology but in the intelligent design of multi-level diagnostic pathways. A first level of field screening, broad and low-cost (for example, with counting apps for *Varroa* or symptomological observations), can serve as an early warning network. Suspected cases identified activate a second level of apiary confirmation, with medium-cost and rapid tools such as LAMP kits. Only for the most complex situations or for basic research would one finally resort to a third level of high-resolution laboratory analysis, such as multiplex qPCR or sequencing. This pyramidal model optimizes resource use, directing analytical complexity and cost only where strictly necessary, and represents the most mature practical application of the principles of rigorous yet sustainable apistic science.

In conclusion, this integrated decision framework shifts the paradigm from searching for the best method in absolute terms to identifying the optimal method for a context. It makes explicit the inevitable trade-offs (cost vs. accuracy, speed vs. precision, practicality vs. completeness) and provides a logical grid to navigate them. Only by embracing this complexity and replacing generic recipes with structured and transparent decision-making processes can the apistic community (from basic research to field application) maximize the effectiveness of resources deployed in the fight against parasites.

## 8. The Role of Geographic Information Systems

The epidemiology of apistic diseases possesses an intrinsically spatial dimension, often undervalued in traditional study designs. Foraging bees operate within an average radius of 3–5 km from the hive (up to 12 km under resource scarcity conditions) [70,71], exposing the colony to risk factors distributed irregularly across the landscape: nectar sources contaminated by pesticides, aggregation areas for drift or robbing, apiary clusters with high parasitic pressure, or refuge zones for wild species serving as pathogen reservoirs [72]. This spatial connectivity implies that a colony’s health cannot be understood in isolation but as part of a territorial ecological network. Geographic Information Systems (GIS) and spatial analysis techniques represent powerful tools to quantify these relationships, identify non-random patterns, and support management decisions at the regional or national scale [73].

### 8.1. Spatial Patterns in Apistic Pathologies

Recent studies on georeferenced data from national surveys (e.g., COLOSS [66]) systematically reveal spatial autocorrelation: nearby colonies present health profiles more similar than predicted by chance alone [73,74]. This clustering is evident for *Varroa destructor*, where adjacent apiaries share infestation levels positively correlated up to distances of 10–20 km, reflecting dispersal through drift, robbing, or human movement [75,76,77]. Similarly, *Vairimorpha ceranae* prevalence shows regional hotspots linked to humid microclimates or agricultural intensity [78]. Landscape factors such as percentage of intensive agricultural cover (correlated with pesticide exposure) or habitat fragmentation (reduced floristic diversity) explain up to 30–50% of spatial variance in colony losses [79].

### 8.2. Identification of Clusters and Hotspots

GIS techniques permit application of rigorous spatial statistical tests to distinguish real aggregations from random fluctuations. Global methods such as Moran’s I index quantify overall autocorrelation, while local approaches (Getis-Ord Gi, Kulldorff’s scan statistics) identify specific hotspots—areas with significantly elevated prevalence [80]. For example, analyses on Italian data post-*Aethina tumida* introduction revealed coastal clusters in Calabria and Sicily, guiding targeted eradication interventions [81]. For *Tropilaelaps* spp., spatial risk models integrate importation data (ports/airports) with climatic suitability, predicting potential introduction corridors [82].

### 8.3. Surveillance and Early Warning Systems

Integration of GIS into surveillance platforms (e.g., COLOSS initiatives) enables near-real-time monitoring. Data voluntarily shared by beekeepers (*Varroa* levels, treatments, losses) are georeferenced and visualized on interactive dashboards, allowing early identification of emerging outbreaks. This approach has demonstrated effectiveness in tracking the spread of acaricide resistance in North America, with dynamic maps highlighting East–West gradients [83]. Challenges include data privacy (georeferencing anonymization) and incomplete coverage in rural areas, but incentives (personalized local risk reports) are increasing participation.

### 8.4. Predictive Models and Future Scenarios

Advanced spatial models, such as MaxEnt or geostatistical machine learning (spatial random forests), combine environmental layers (climate, land use, apiary density) with epidemiological data to predict risk in unsampled areas [84]. Projections under climate scenarios indicate northwestern expansion of suitable zones for *Tropilaelaps* in Europe, with uncertainties quantified through bootstrap confidence intervals. Similarly, models for *Varroa* predict an increase in infestation intensity in temperate regions due to additional reproductive cycles [85,86]. These tools support adaptive planning: positioning new apiaries in predicted low-risk zones or prioritizing surveillance in vulnerable corridors.

### 8.5. Limitations and Perspectives

Despite progress, limitations persist: data quality (voluntary reporting bias), resolution scale of environmental layers, and model complexity requiring independent validation. Future developments include integration with remote-sensed data (drone/satellite for flowering mapping) and georeferenced citizen-science platforms. A spatial One Health approach, including wild reservoirs (bumblebees, solitary bees), will further amplify potential.

## 9. Emerging Technologies in Apistic Diagnostics

Emerging technologies (from artificial intelligence (AI) for automatic *Varroa* counting [87], to isothermal LAMP kits for *Vairimorpha* [30], to integrated continuous monitoring systems) promise to revolutionize apistic diagnostics, making it faster, more scalable, and accessible. However, a critical evaluation of recent literature reveals that many of these innovations are still transitioning from laboratory to field, with real-world performance often lower than that claimed under controlled conditions. Enthusiasm must be tempered by rigorous analysis of accuracy, robustness, total costs, and operational limitations to avoid premature adoption that could generate false senses of security.

### 9.1. Image-Based Automation and Artificial Intelligence

Smartphone applications and AI systems for *Varroa* counting from photographs of bees on white trays represent one of the most visible advances. Initial laboratory studies reported high accuracies (>95%) [87]. However, a critical evaluation reveals a significant gap between controlled validation and field deployment. While algorithm performance under ideal conditions can be robust, real-world performance is frequently compromised by environmental confounders, with sensitivity dropping 10–20% in humid or debris-heavy conditions [87]. This often leads to a notable drop in sensitivity, particularly in samples with low infestation levels, which risks delaying critical interventions. Furthermore, these vision-based systems are inherently limited to the phoretic phase on adult bees. They are blind to mites in the reproductive phase within sealed brood, which is crucial for understanding infestation growth. For this hidden but critical aspect, novel non-invasive alternatives are emerging, such as accelerometer-based systems that analyze the unique vibrational signatures of mite gait within cells [88]. Consequently, the future of automated monitoring likely lies not in a single technology but in a sensor fusion approach. An integrated system would combine data streams from visual AI, vibrational/acoustic sensors, and environmental IoT platforms (e.g., scales, internal temperature/humidity sensors) to provide a comprehensive, real-time assessment of colony health and parasitic pressure [89]. Therefore, while current AI tools are valuable for rapid, non-lethal screening in routine monitoring [87], they should be considered complementary tools that require confirmation with traditional methods (e.g., ethanol wash) for definitive therapeutic decisions or efficacy trials, and their results must be interpreted with an awareness of their technical limitations.

### 9.2. Isothermal Amplification Techniques (LAMP) and Point-of-Care Diagnostics

LAMP kits for *Vairimorpha* spp. and, more recently, for *Lotmaria passim*, offer species-specific identification in 40–60 min without a thermocycler, with sensitivity comparable to conventional PCR (~10^2^–10^3^ spores/bee) [30]. Their democratization is evident: reduced per-sample costs and visual results facilitate use by beekeeping associations. Persistent limitations include susceptibility to inhibitors in unpurified samples (fecal residues, pollen) and difficulties in robust multiplexing for multi-pathogen panels. Recent field validations indicate false negative rates up to 10–15% in complex samples, making LAMP ideal for preliminary screening but not for definitive confirmation in low-prevalence contexts or mandatory notifications.

### 9.3. Continuous Monitoring and Integrated Sensors

“Connected hive” systems combine scales, microphones, thermal and humidity sensors with AI algorithms to infer infestations (e.g., *Varroa* drop from acoustic patterns or weight variations) [90,91]. European and North American pilots show promising correlations with traditional counts [87], offering non-invasive monitoring and temporal trends. Operational challenges are significant: reliability compromised by weather events, propolis/wax accumulation on sensors, dependence on internet connectivity, and stable power—critical factors in remote apiaries. Moreover, algorithms often operate as “black boxes,” complicating alarm interpretation. High initial costs limit accessibility, risking a divide between professional and hobbyist beekeepers [92].

### 9.4. Advanced Molecular Diagnostics and eDNA

Environmental DNA (eDNA) extraction from bottom board debris or honey promises non-invasive detection of *Varroa* and pathogens [93]. High sensitivity in controlled trials, but limited field validation highlights cross-contaminations and quantitative difficulties. Portable multiplex qPCR is emerging, but disposable reagent costs remain prohibitive for extensive routine use.

## 10. Climate Change and Impacts on Parasitic Dynamics: An Integrated and Evidence-Based Perspective

Climate change represents one of the most powerful and pervasive drivers of health dynamics in apiculture, acting not only as an amplifier of existing risks but also as a catalyst for new epidemiological scenarios. Its influences manifest through thermal, hydrological, and phenological alterations that directly modify parasite biology, host physiology, and ecosystem interactions [85]. This section integrates recent empirical evidence with validated predictive models, avoiding unsupported speculation, to provide a critical evaluation of expected impacts and diagnostic-epidemiological adaptation strategies. The objective is to transform climate projections into concrete operational implications for surveillance and management.

### 10.1. Geographic Expansion of Suitable Areas for Invasive Parasites

Parasites such as *Tropilaelaps* spp. and *Aethina tumida* are currently limited by climatic barriers, but bioclimatic models based on IPCC scenarios predict their potential range expansion; a testable hypothesis is that +2 °C increases *Varroa* reproductive cycles by 20–40%, verifiable via cohort studies across thermal gradients [44]. These projections do not imply automatic establishment but increase introduction risk via commerce and apistic migration, making hierarchical surveillance essential: visual inspections and sentinel traps as first level, followed by molecular confirmation (PCR-RFLP or cox1 sequencing).

Similarly, for *Aethina tumida*, Mediterranean regions (southern Italy) are already at the edge of suitability; a 1–2 °C increase in minimum temperatures could extend permanent risk areas, with models incorporating relative humidity and precipitation estimating a 20–35% expansion [94]. Here, soil sampling for pupae remains complementary to in-hive traps, but diagnostics must prioritize non-destructive methods for extensive monitoring.

### 10.2. Alterations in Reproductive Dynamics and Virulence in Endemic Parasites

For *Varroa destructor*, reproductive rate is temperature-dependent: data from warm climates (e.g., subtropical regions) show additional cycles per season with +2 °C, leading to a potential 20–40% increase in parasitic population (with variations based on local genotypes) [95]. This accelerates autumn peaks, reducing the window for interventions [96]. Moreover, elevated temperatures favor replication of vectored viruses, with viral loads increasing exponentially above 30 °C [97]. Diagnostics must adapt: continuous monitoring (natural drop with seasonal corrections) integrated with qPCR for DWV in bee pools, accounting for apiary-specific clustering through mixed models.

*Vairimorpha ceranae*, thermophilic compared to *Vairimorpha apis*, benefits from prolonged summers: prevalences may increase 15–30% under warming scenarios, with seasonal patterns shifting toward summer peaks [98]. Synergies with *Lotmaria passim* are exacerbated by thermal stress, which alters the intestinal microbiome [99]. Multiplex protocols (qPCR or LAMP) become priorities to capture co-infections.

### 10.3. Implications for Epidemiology and Diagnostics

These changes require a methodological evolution, structured around three key actions: (1) in field studies, longitudinal cohort studies must incorporate climatic variables (e.g., degree-days) and use hierarchical models to account for intraclass correlation among differently exposed apiaries; (2) in data synthesis, it is necessary to integrate real-time climatic layers (from Copernicus or NOAA) with prevalence data for dynamic risk maps, identifying outbreak clusters with spatial statistics (e.g., Getis-Ord Gi); (3) in the “Context” pillar, climate projections should be included to guide method selection (e.g., preferring non-lethal techniques in high-risk areas to preserve potentially resilient colonies).

While uncertainties persist (e.g., regarding local parasitic adaptation), evidence from current climatic gradients (such as between Northern vs. Southern Europe) strongly supports these trajectories.

## 11. Ethical, Biosecurity, and Colony Welfare Considerations: An Integrated and Contextualized Approach

Apistic research and practice operate in a complex ethical context, where diagnostic and epidemiological decisions must balance knowledge advancement with minimization of harm to colonies, protection of pollinator biodiversity, and prevention of biosecurity risks. Traditionally focused on vertebrates, ethical principles increasingly apply to invertebrates, recognizing bees as super-organisms with collective sentient capacities (e.g., stress responses, colonial homeostasis). This section critiques current applications of the 3Rs (Replacement, Reduction, Refinement), integrates biosecurity considerations, and introduces the concept of colony-level welfare, proposing practical adaptations to the decision framework.

### Application of 3Rs Principles in Apistic Research

The 3Rs principles, originally developed for vertebrate research (Russell & Burch, 1959) [100], find growing application in invertebrate studies, supported by EU guidelines (Directive 2010/63/EU, extended to cephalopods but with ethical recommendations for social insects). In the apistic context:

Replacement: substitution of in vivo models with non-animal alternatives. Examples include in silico models for parasitic dynamics (e.g., *Varroa* population simulations) [101]. Cell cultures of apistic lines (e.g., AmE-711 for *Vairimorpha* studies) reduce the need for experimental infections [102]. Metagenomics on historical samples or environmental eDNA substitutes destructive sampling for surveillance.

Reduction: minimization of the number of colonies/bees used while maximizing information per unit. Efficient experimental designs (e.g., hierarchical mixed models for accounting apiary-specific clustering, ICC 0.2–0.4) reduce sample size by 20–40% without power loss (calculations based on prevalence formula with design effect correction). Pooled sampling (e.g., 30–50 bees for multiplex qPCR) and non-invasive monitoring (acoustic sensors, scales) optimize longitudinal data [11,103].

Refinement: perfection of procedures to minimize stress and suffering. Non-lethal methods (powdered sugar vs. ethanol wash) are preferable for routine monitoring; when destructive (e.g., brood dissection), justified only for high-precision objectives (efficacy trials) [19]. Euthanasia: gradual freezing (−20 °C) or CO_2_, per EU bee welfare guidelines [104]. Remote monitoring reduces manipulations, preserving natural behavior.

Bees are not isolated individuals but super-organisms; therefore, their welfare must be evaluated at the colony level. This assessment relies on indicators such as productivity, resilience, and foraging behavior. However, invasive diagnostic methods (e.g., destructive brood sampling) negatively impact the collective fitness of the colony. Their use is ethically justifiable only through an explicit trade-off between the benefit of knowledge and the cost to the super-organism. The application of the 3Rs principle (Replace, Reduce, Refine) in beekeeping remains inadequate, as its traditional focus on the individual must be adapted to the colony unit. Recent proposals aim to address this gap by defining specific ‘colony welfare indicators,’ such as brood pattern regularity and reserve accumulation [105].

## 12. Knowledge Gaps and Agenda for Future Research: Toward Integrated and Resilient Diagnostics

Despite substantial progress in understanding apistic parasites and in diagnostic and epidemiological methodologies, significant gaps persist that limit the capacity to effectively manage these threats in a rapidly evolving global context. This section critically identifies such gaps, based on a synthesis of recent literature and systematic analyses, to propose a priority research agenda. The objective is not mere enumeration but formulation of testable hypotheses and methodological approaches that can fill these gaps, emphasizing originality through integration of global perspectives, rigorous application of epidemiological frameworks (such as Hill’s criteria), and empirical validation of the decision-making framework proposed in this review.

### 12.1. Multi-Pathogen Synergies and Complex Interactions

One of the most critical gaps concerns quantitative understanding of synergistic interactions among parasites, pathogens, and other stressors (viruses, bacteria, pesticides, malnutrition) [106]. Although studies such as Alaux et al. (2010) indicate interactions between *Vairimorpha* and neonicotinoids [107], and recent analyses quantify 30–40% increases in mortality for *Lotmaria passim-Vairimorpha ceranae* co-infections [61], a systematic evaluation applying Hill’s criteria to infer causality in real contexts is lacking. For example, temporality and dose–response gradient are often inferred from laboratory studies but not validated in large-scale longitudinal cohorts.

In this context the conduction of multi-year prospective cohort studies (at least 3–5 years) in different biogeographic regions (temperate Europe, Mediterranean, Americas, Africa with Africanized bees), integrating multiplex molecular panels (qPCR for *Vairimorpha* spp., *Lotmaria passim*, *Crithidia mellificae*, DWV and other key viruses) with gut microbiome metagenomics become a priority. These studies should incorporate hierarchical mixed models for accounting for apiary-specific clustering, proposing meta-analyses to synthesize global evidence and identify contextual variations (e.g., tolerance in Africanized bees).

### 12.2. Field Validation of Emerging Technologies and Hierarchical Integration

Emerging technologies (AI for *Varroa* counting [87], point-of-care LAMP [29], IoT sensors) promise diagnostic democratization, but studies highlight accuracy drops from lab to field, with critical false negatives due to inhibitors or environmental variability. Independent validation on a global scale is lacking, including false positives in qPCR (from non-viable spores) and GIS limitations (data privacy, reporting bias).

Multicentric randomized controlled trials (RCTs) to test the effectiveness of the four-pillar decision framework (Section 7) in selecting hierarchical methods (AI/LAMP screening → traditional confirmation) are necessary. Hierarchical approach could reduce costs by 30–50%, maintaining sensitivity > 95% in resource-limited contexts (e.g., African vs. European beekeeping).

### 12.3. Climate Change Impact and Predictive Models

Speculative projections about emerging parasite expansion lack precise empirical references and quantified uncertainty. Data from warm climates show variable *Varroa* adaptation but are not integrated with viral replication or *Tropilaelaps-Varroa* co-occurrence in Asia. The development of integrated predictive models that combine bioclimatic and epidemiological factors, validated against long-term historical data sets such as those from COLOSS surveys [66], holds significant promise for clarifying these complex interactions. A central, testable hypothesis is that rising temperatures may accelerate key *Varroa destructor* reproductive parameters, a relationship that can be quantified using robust empirical data from natural thermal gradients and expressed with empirically derived confidence intervals. To ensure global relevance, these models should incorporate comparative data on regional host–parasite dynamics (including the notable tolerance found in Africanized bee populations) and employ GIS frameworks to generate dynamic risk maps. Furthermore, adopting a One Health perspective is crucial for projecting broader ecological consequences, particularly the cascading effects on wild pollinator communities.

## 13. Conclusions

The evidence synthesized here suggests that effective management of apistic parasites may require a paradigm shift, moving from the search for universal solutions towards the principle of optimal contextual choice. As demonstrated by critical analysis of methods for *Varroa*, *Vairimorpha*, and other pathogens, effectiveness depends on the capacity to select and integrate tools based on specific objectives, resources, and scenarios. The proposed decision framework, founded on the pillars of objective, constraints, context, and ethics, provides the logical grid to translate this principle into operational action.

Future efforts should prioritize research on multi-pathogen interactions, climate change impacts, and sustainable control development. However, real progress will depend on the community’s capacity to collaborate, share data, and adopt a practical One Health approach, recognizing bee health as an ecological and economic common good.

## Figures and Tables

**Table 1:** Comparative overview of main diagnostic methods for *Varroa destructor* monitoring.

Method	Sensitivity (%)	Specificity (%)	Time (min)	Cost per Sample (€)	Advantages	Limitations	Optimal Scenario	Update
Powdered sugar roll [18]	75–90	~100	10–15	0.10–0.50	Non-lethal; rapid; bees reusable; low cost	Variable sensitivity; stressful for bees; influenced by humidity/temperature	Rapid field monitoring; beekeepers; non-lethal longitudinal studies	AI apps for automatic counting
Ethanol wash [18]	98–100	100	20–30	1.00–2.00	Maximum accuracy; sample preservation; reference standard	Lethal; requires ethanol; sample disposal	Research requiring precise counts; validation of other methods; efficacy trials	Confirmed as reference standard
Natural mite fall [18]	80–95	100	Continuous monitoring	0.05–0.20 (per day)	Non-invasive; continuous monitoring; good correlation with infestation	Variable correction factors (brood vs. broodless); time delay	Seasonal monitoring; trend assessment; early warning in extensive beekeeping	AI integration for automatic counting
Brood sampling [18]	90–100 (per cell)	100	30–45	0.50–1.50	Provides data on mite reproduction; estimates total population	Destructive to brood; labor-intensive; requires experience	Studies on reproductive biology; assessment of brood damage; model calibration	Useful for eDNA detection
Total acaricide [18]	~100	~100	Weeks	5.00–10.00	Measures overall post-treatment efficacy; assesses residual mite load	Long time; does not provide dynamic data; high cost	Treatment efficacy evaluation	Combined with molecular tests to monitor resistance; rising resistance monitored

**Table 2:** Comparison of diagnostic technologies for the detection and identification of *Vairimorpha* spp.

Technology	Detection Threshold	Species-Specific Identification	Quantification Capability	Accessibility (Cost/Equipment)	Standardization Level	Main Knowledge Gap/Limitation
Optical Microscopy	~10^4^ spores/bee	No (morphological only)	Semi-quantitative	High (low cost, common equipment)	Moderate (inter-observer variability)	Distinguishing *Vairimorpha apis*/*Vairimorpha ceranae* impossible in many cases; low sensitivity
Conventional PCR	~10^2^–10^3^ spores/bee	Yes (with specific primers)	No (qualitative)	Medium (basic molecular equipment)	Low (variable protocols)	No quantification; risk of false negatives with low pathogen loads
Real-time qPCR	~10^1^–10^2^ spores/bee	Yes (with specific probes/primers)	Yes (absolute or relative)	Low (high cost, specialized equipment)	Medium (requires calibrated standards)	Non-linear conversion of genomic copies → viable spores; high cost
LAMP (Isothermal)	~10^2^–10^3^ spores/bee	Yes (high specificity)	Semi-quantitative (visual)	Medium–High (commercial kits, thermal block)	Increasing (validated kits available)	Limited multiplexing; potential inhibitors in complex samples
Sequencing (NGS)	~10^1^–10^2^ spores/bee	Yes (maximum resolution, detects strains)	Yes (relative metagenomics)	Low	Medium	High cost; complex data analysis

**Table 3:** Characteristics and applications of the main epidemiological study designs in apicultural research.

Study Type	Strength of Evidence (Causality)	Relative Cost	Time Duration	Logistic Complexity	Example of Application	Main Source of Bias to Control
Cross-sectional	Low (association)	Low	Short (one time point)	Low	Estimating the prevalence of *Vairimorpha* in a region	Cause/effect not determinable; participant selection
Case–control	Medium–High	Medium	Short (retrospective)	Medium	Identifying risk factors for colony collapse	Recall bias; selection of appropriate controls
Cohort	High	High	Long (prospective)	High	Evaluating the impact of a new acaricide on winter mortality	Loss to follow-up; changes in practices over time
Randomized Controlled Trial (RCT)	Maximum	Very High	Long	Very High	Testing the efficacy of a nutritional supplement	Hawthorne effect; difficulty in blinding
Ecological studies	Very Low	Very Low	Variable	Very Low (often uses existing data)	Comparing regional pesticide use with apiary loss trends	Ecological fallacy (group-level data vs. individual risk)

## Data Availability

No new data were created in this study.
